# Irisin Gene Delivery Ameliorates Burn-Induced Sensory and Motor Neuropathy

**DOI:** 10.3390/ijms21207798

**Published:** 2020-10-21

**Authors:** Shu-Hung Huang, Shih-Ming Yang, Jing-Jou Lo, Sheng-Hua Wu, Ming-Hong Tai

**Affiliations:** 1 Division of Plastic Surgery, Department of Surgery, Kaohsiung Medical University Hospital, Kaohsiung 807, Taiwan; huangsh63@gmail.com; 2 Department of Surgery, School of Medicine, College of Medicine, Kaohsiung Medical University, Kaohsiung 807, Taiwan; 3 Regeneration Medicine and Cell Therapy Research Center, Kaohsiung Medical University, Kaohsiung 807, Taiwan; 4 Graduate Institute of Medicine, College of Medicine, Kaohsiung Medical University, Kaohsiung 807, Taiwan; 5 Institute of Biomedical Sciences, National Sun Yat-Sun University, Kaohsiung 804, Taiwan; yshihming@gmail.com; 6 Department of General Medicine, Kaohsiung Medical University Hospital, Kaohsiung Medical University, Kaohsiung 807, Taiwan; ljingjou@gmail.com; 7 Department of Anesthesiology, Kaohsiung Medical University Hospital, Kaohsiung 807, Taiwan; 8 Department of Anesthesiology, School of Medicine, College of Medicine, Kaohsiung Medical University, Kaohsiung 807, Taiwan; 9 Department of Anesthesiology, Kaohsiung Municipal Ta-Tung Hospital, Kaohsiung 801, Taiwan

**Keywords:** irisin, apoptosis, neuroinflammation, burn injury, neuropathy

## Abstract

Burn-related neuropathy is common and often involves pain, paresthesia, or muscle weakness. Irisin, an exercise-induced myokine after cleavage from its membrane precursor fibronectin type III domain-containing 5 (FNDC5), exhibits neuroprotective and anti-inflammatory activities. A rat model of third-degree burn on the right hind paw was used to investigate the therapeutic role of irisin/FNDC5. Rats received burn injury and were treated with intrathecal recombinant adenovirus containing the irisin sequence (Ad-irisin) at 3 weeks postburn. One week later, mechanical allodynia was examined. The expression of irisin in cerebrospinal fluid (CSF) was detected. Ipsilateral gastrocnemius muscle and lumbar spinal cord were also obtained for further investigation. Furthermore, the anti-apoptotic effect of recombinant irisin in SH-SY5Y cells was evaluated through tumor necrosis factor alpha (TNFα) stimulus to mimic burn injury. We noted intrathecal Ad-irisin attenuated pain sensitization and gastrocnemius muscle atrophy by modulating the level of irisin in CSF, and the expression of neuronal FNDC5/irisin and TNFα in the spinal cord. Ad-irisin also ameliorated neuronal apoptosis in both dorsal and ventral horns. Furthermore, recombinant irisin attenuated TNFα-induced SH-SY5Y cell apoptosis. In summary, irisin attenuated allodynia and muscle wasting by ameliorating neuroinflammation-induced neuronal apoptosis.

## 1. Introduction

Neuropathy following burn trauma is common and often undiagnosed [1,2,3]. According to the WHO report, nearly 11 million severe burn injuries occur annually worldwide [4]. Up to 52% of burn patients develop neuropathy [5,6]. Patients with burn-related neuropathy often experience pain, paresthesia, or muscle weakness [2,7]. These long-term sequelae negatively affect quality of life and can be challenging to manage, which largely impact economic and social consequences on the patient, family, and society [8]. Increased production of inflammatory cytokines, such as tumor necrosis factor alpha (TNFα) [9,10,11], initiates these complex neuromuscular and neurosensory complications. Patients with burn often experience severe acute pain, and approximately half of these patients develop persistent pain for several years, even beyond the wound healing period [5,6], which can be characterized as sensory neuropathy with spontaneous pain or hyperalgesia over the postburn area. Our previous investigation indicated that neuroinflammation, autophagy, and neuronal apoptosis in the dorsal horn following burn contributed to burn-induced neuropathic pain [12]. Several treatments have been applied for chronic pain, such as gabapentinoids, antidepressants, steroids, and opioids, but they have insufficient efficacy, and their adverse effects limit their usage [13,14]. In addition, burn-induced motor neuropathy, typically manifesting with muscle weakness, is also noted [1,15]. However, the underlying mechanism remains unclear. An experimental full-thickness burn model indicated a reduction of conduction velocities postburn [16]. Our previous study on a burn rat model also indicated increased apoptosis in the spinal cord ventral horn postburn, resulting in denervation muscle atrophy [17]. Ma et al. further indicated that microglial activation in the ventral horn resulted in neuronal apoptosis [18]. Therefore, an effective agent against burn-related neuropathic complications is required.

Irisin, an exercise-induced hormone, is cleaved from its precursor fibronectin type III domain-containing 5 (FNDC5) and mainly secreted by skeletal muscle in response to physical activity [19,20]. Irisin was primarily reported to function as a myokine with thermogenetic capacity that promotes adipocyte browning and was suggested to improve metabolic conditions, including insulin resistance, type 2 diabetes, and obesity [19,21]. However, growing evidence suggests that it acts on multiple tissue types [22] and exerts, among others, neuroprotective and anti-inflammatory effects [23,24]. In a middle cerebral artery occlusion mouse model, irisin was reported to protect against cerebral ischemia-induced neuronal injury by reducing oxidative stress, suppress inflammation by reducing plasma TNFα and IL-6, inhibit the activation of Iba1^+^ microglia, and infiltrate MPO-1^+^ monocytes [25]. Irisin inhibits not only microglial activation but also NF-κB activation in cultured astrocytes, thereby reducing downstream COX-2 expression and releasing IL-6 and IL-1β [26]. Another study demonstrated its neuroprotective effect in cerebral ischemia/reperfusion injury by irisin-induced reduction in the activation of apoptosis and TNFα and IL-1β expression in brain tissue [27]. Inflammation contributes to not only neuronal damage but also the pathogenesis of nociceptive and neuropathic pain [28,29]. Inflammatory mediators released by immune cells, such as TNFα, IL-1β, IL-6, PGE_2_, and PGI_2_, result in pain sensitization [28]. Exercise and physical therapy alleviate chronic neuropathic pain by modulating neuroinflammation [29,30,31,32,33]. Irisin has also been suggested to alleviate pain sensitization [34,35] and possess diagnostic value for pain symptoms [36,37]. However, its role in burn-induced neuropathy remains unclear.

We previously reported that third-degree burn injury caused neuronal apoptosis in both ventral and dorsal horns, resulting in denervation muscle atrophy and neuropathic pain up to 4–8 weeks, respectively [12,17,38]. To elucidate the potential role of irisin in neuroprotection, we administered intrathecal irisin gene delivery in a third-degree burn rat model. Mechanical allodynia was tested following injection. FNDC5/irisin and TNFα expression and neuronal apoptosis in both the dorsal and ventral horns were examined. Survival markers of motor neuron in the ventral horn and gastrocnemius muscle atrophy were also evaluated. Furthermore, the antiapoptotic effect of recombinant irisin on neuronal cells was investigated with TNFα stimulus to mimic burn injury in vitro.

## 2. Results

### 2.1. Expressions of Spinal FNDC5/Irisin and CSF Irisin Were Decreased after Burn Injury

**Figure 1:**
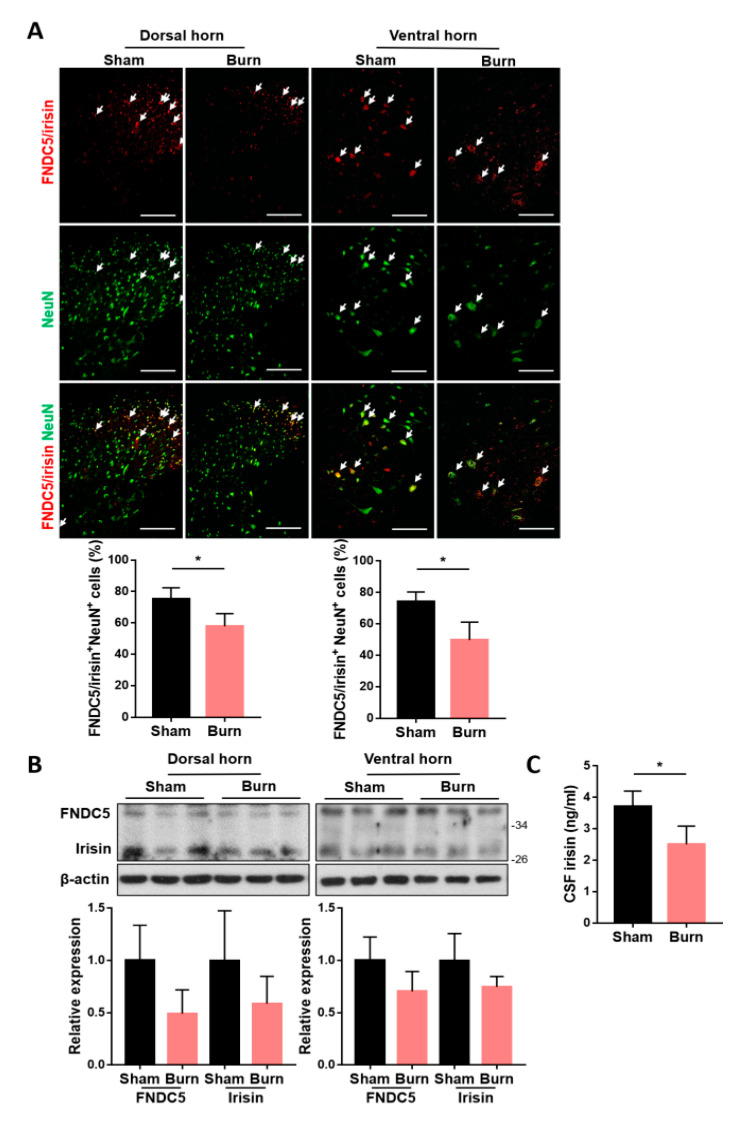
The expression of fibronectin type III domain-containing 5 (FNDC5)/irisin in the ventral and dorsal horns was reduced after burn injury. (**A**) Immunofluorescence staining of FNDC5/irisin and NeuN in the lumbar spinal cord. Representative bar graph illustrating the ratio of double-positive cells to NeuN^+^ cells. Error bars, mean ± SD. * *p* < 0.05, unpaired *t* test. Scale bar: 100 μm. (**B**) Immunoblotting analysis for the expression of FNDC5 and irisin in the dorsal (left panel) and ventral (right panel) horns. Representative bar graph illustrating the normalized expression ratio of FNDC5 and irisin with β-actin. (**C**) Bar graph illustrating the irisin concentration in cerebrospinal fluid (CSF) obtained using an enzyme-linked immunosorbent assay. Error bars, mean ± SD. * *p* < 0.05, unpaired *t*-test.

To identify whether spinal expressions of FNDC5/irisin were affected by burn injury, we introduced third-degree burn on the right hind paw of each rat [12,17,38]. The ipsilateral spinal cord of L4–L6 segments corresponding to innervation on the hind limb [39] and cerebrospinal fluid (CSF) were obtained at 4 weeks postburn. The lumbar segments were subjected to immunofluorescence staining and immunoblotting analysis for FNDC5/irisin expression. We found that burn injury resulted in a decrease in FNDC5/irisin^+^ cells in both dorsal and ventral neuronal NeuN^+^ cells (Figure 1A). Moreover, immunoblotting analysis indicated a trend of downregulation in both FNDC5 and irisin expression (Figure 1B). The CSF irisin level was decreased in the burn group (Figure 1C). In addition, burn injury reduced the expression of NeuN^+^ cells in both the dorsal and ventral horns (Figure 1A). These results indicated that burn injury causes neuronal damage in sensory and motor neurons postburn and that precursor FNDC5 expression and irisin secretion might be involved in the survival of neurons in the spinal cord.

### 2.2. Adenoviral Vector Design for Irisin Gene Delivery and Validation of Recombinant Irisin Secretion

**Figure 2:**
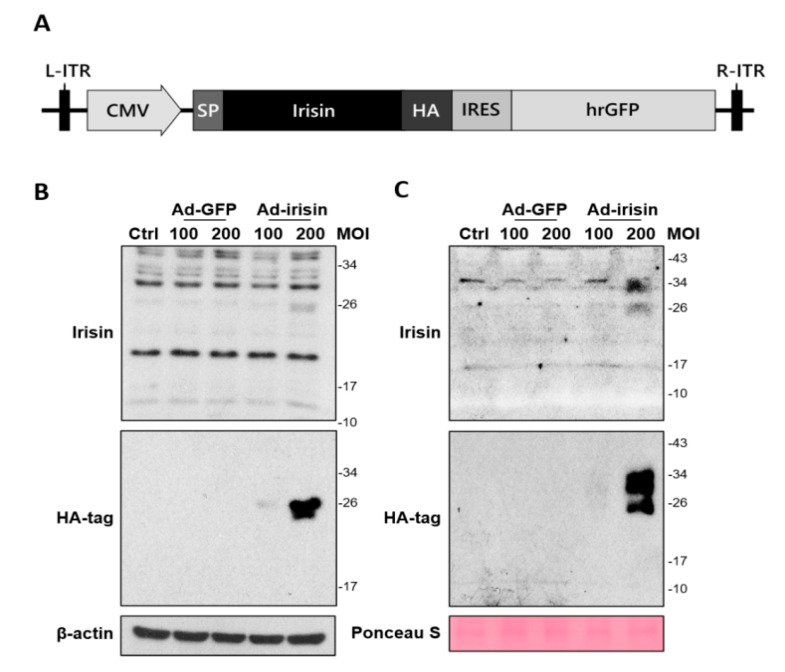
Generation and validation of adenovirus-mediated irisin expression in cultured astrocytes. (**A**) A vector for generating adenovirus containing the irisin sequence (Ad-irisin) was designed using an AdEasy adenoviral vector system (Agilent Technologies, CA, USA). The mouse irisin gene containing an N-terminal signal sequence was subcloned into multiple cloning sites on the pShuttle-IRES-hrGFP-2 vector. After recombination with the pAdEasy-1 vector in competent cells, the pShuttle-irisin-IRES-hrGFP-2 vector was isolated and transfected into HEK293 cells to produce Ad-irisin. Vector without insertion was used to generate Ad-GFP. C6 cells were infected with Ad-irisin at 100 and 200 MOI for 48 h with a supplement of 10% FBS. Infection with Ad-GFP was used as an infectious control. (**B**) Cell extract and (**C**) culture medium were collected and subjected to immunoblotting for irisin expression and HA-tag signal.

To further investigate whether irisin expression and secretion exerted neuroprotection, we manipulated the generation of an adenoviral system for irisin gene delivery. In brief, a recombinant adenoviral vector containing an intact irisin gene with an N-terminal signal sequence was transfected to HEK293 cells to produce adenoviral particles (Figure 2A). The adenoviral particles were then infected with C6 rat glioma cells for validation of secretion. Cellular expression and release of recombinant irisin after infection at 48 h was validated using immunoblotting. The results revealed that C6 strongly expressed recombinant irisin (approximately 26 kDa) at 200 MOI with adenovirus containing the irisin sequence (Ad-irisin) infection (Figure 2B). The secretion of recombinant irisin was validated by analysis in a culture medium (Figure 2C). In addition, a portion of recombinant irisin exhibited an increased molecular weight in the culture medium (Figure 2C) compared with the cell extract (Figure 2B), and this result was validated using a specific anti-HA-tag antibody (Figure 2B,C). Thus, we validated the infectivity, expression, and secretion of recombinant irisin through our adenoviral system. In addition, the increased molecular weight of recombinant irisin suggests unknown modifications for secretion, which requires further investigation.

### 2.3. Intrathecal Irisin Gene Delivery Alleviated Postburn Allodynia

**Figure 3:**
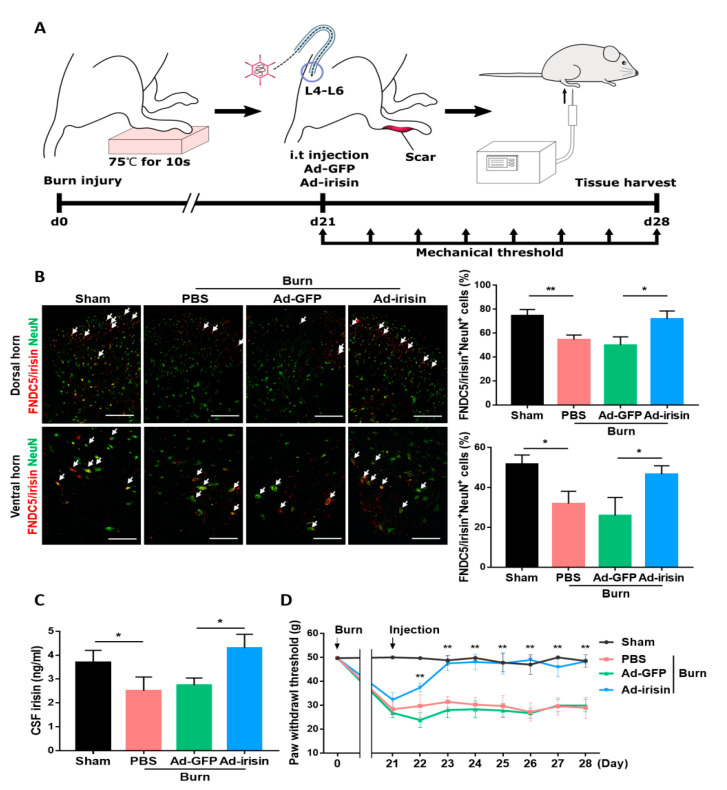
Intrathecal irisin gene delivery restored spinal irisin expression and alleviated burn-induced neuropathic pain. (**A**) Experimental scheme of our third-degree burn rat model. Third-degree burn injury was introduced on day 0 with 75 ± 0.5 °C for 10 s. Intrathecal Ad-GFP and Ad-irisin (1 × 10^8^ CFU) were injected at 3 weeks postburn. The mechanical threshold was measured before treatment and each day following treatment till 4 weeks postburn. (**B**) Double immunofluorescence staining of FNDC5/irisin and NeuN in the dorsal and ventral horns of L4–L6 segments at 4 weeks postburn. Arrowheads indicate double-positive cells. Representative bar graph illustrating the ratio of double-positive cells to NeuN^+^ cells. Error bars, mean ± SD. * *p* < 0.05, unpaired *t* test. Scale bar: 100 μm. (**C**) Bar graph representing cerebrospinal fluid irisin measured using an enzyme-linked immunosorbent assay. Error bars, mean ± SD. * *p* < 0.05, unpaired *t* test. (**D**) Paw withdrawal test. Average von flay pressures are shown. Error bars, mean ± SD. ** *p* < 0.01 represents the Ad-irisin group compared with the Ad-GFP group, unpaired *t*-test.

To determine whether the spinal gene delivery of irisin can alleviate neuronal damage and pain sensitization, we administered intrathecal Ad-irisin in our burn rat model (Figure 3A). Ad-irisin was injected at 3 weeks postburn; 1 week later, we confirmed irisin gene delivery as well as increased FNDC5/irisin and NeuN double-positive cells compared with the Ad-GFP group in both the dorsal and ventral horns (Figure 3B). Furthermore, double immunofluorescence staining of irisin and GFAP showed that burn injury resulted in decrease FNDC5/irisin expression in GFAP^+^ cells, whereas an attenuated reduction was found in the Ad-irisin group (Figure S1). The detection of CSF irisin indicated the restoration of basal irisin levels in the Ad-irisin group (Figure 3C), suggesting that our Ad-irisin system was functional both in vitro and in vivo. The paw withdrawal test indicated that intrathecal Ad-irisin alleviated mechanical allodynia (Figure 3D). In addition, after Ad-irisin injection, the number of NeuN^+^ cells increased in both dorsal and ventral horns (Figure 3B), suggesting that irisin might exert neuroprotective effects after distal burn injury.

### 2.4. Irisin Gene Delivery Alleviated Neuronal Apoptosis in the Spinal Cord Postburn

**Figure 4:**
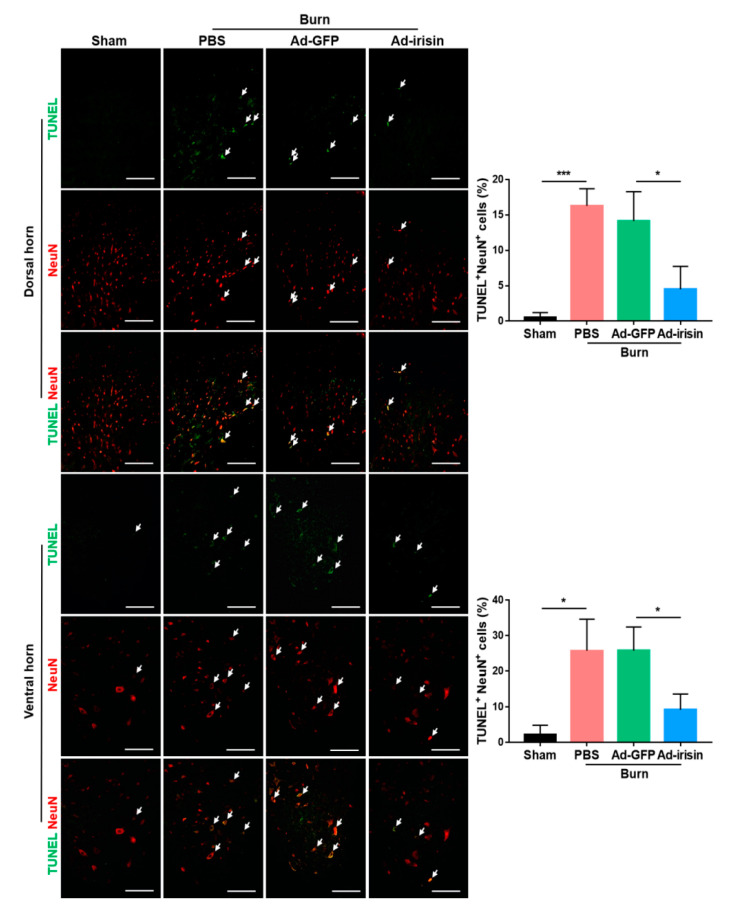
Irisin gene delivery alleviated burn-induced neuronal apoptosis in the dorsal and ventral horns. TUNEL staining and immunofluorescence of NeuN were illustrated. Arrowheads indicate double-positive cells. Representative bar graph illustrating the ratio of TUNEL^+^NeuN^+^ cells to NeuN^+^ cells. Error bars, mean ± SD. * *p* < 0.05, *** *p* < 0.001, unpaired *t*-test. Scale bar: 100 μm.

To evaluate whether irisin gene delivery exerted neuroprotective effects postburn, TUNEL staining was employed to examine apoptotic events in the ipsilateral spinal cord. We found that burn injury resulted in an increased number of apoptotic cells in both the dorsal and ventral horns, whereas intrathecal Ad-irisin injection alleviated apoptosis (Figure 4). These results indicated that increased expression and secretion of irisin protects neuronal cells from apoptotic cell death.

### 2.5. Irisin Improved the Survival of Motor Neuron Cells and Muscle Size Following Burn Injury

**Figure 5:**
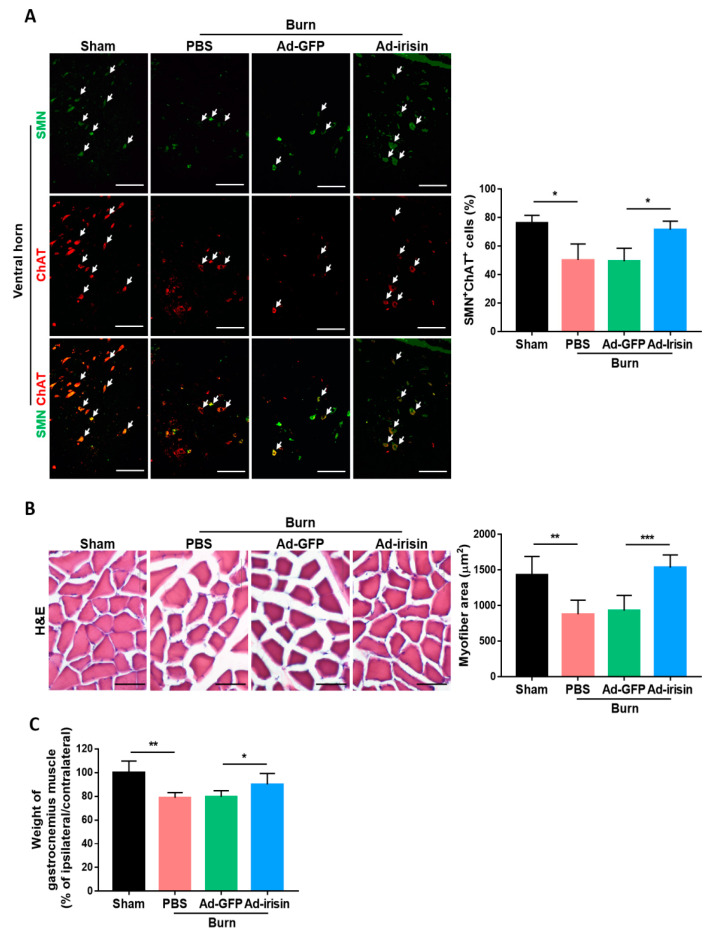
Adenovirus containing the irisin sequence rescued motor neuronal survival in the ventral horn and muscle deterioration after burn injury. (**A**) Double immunofluorescence of survival motor neuron protein (SMN) and choline acetyltransferase (ChAT) in the ventral horn. Arrowheads indicate double-positive cells. Representative bar graph illustrating the ratio of SMN^+^ChAT^+^ cells to ChAT^+^ cells. Error bars, mean ± SD. * *p* < 0.05, unpaired *t* test. (**B**) H&E stain of gastrocnemius muscle. Scale bar 50 μm. Representative bar graph illustrating area of myofiber. Error bars, mean ± SD. ** *p* < 0.01, *** *p* < 0.001, unpaired *t*-test. (**C**) Representative bar graph illustrating the wet weight of gastrocnemius muscle in the ratio of ipsilateral to contralateral site. Error bars, mean ± SD. * *p* < 0.05, ** *p* < 0.01, unpaired *t* test.

To investigate whether irisin exerts a neuroprotective effect in functional motor neurons, we further examined the expression of survival motor neuron protein (SMN), which is required for motor neuron survival and neurite outgrowth [40,41]. Deficiency of SMN in motor neuron is linked to spinal muscular atrophy [42]. Motor neuron-specific SMN knockdown leads to motor neuron degeneration and muscle atrophy [43]. Therefore, we carried out immunofluorescence staining for SMN expression in the ventral horn at 4 weeks postburn. The antibody against choline acetyltransferase (ChAT) staining was used as a general motor neuron marker [44]. The ratio of SMN^+^ cells to ChAT^+^ cells was significantly decreased in the burn group, whereas an attenuated reduction was found in the Ad-irisin group (Figure 5A). Burn injury caused a decrease in the myofiber cross-sectional area by H&E staining (Figure 5B) and wet weight of gastrocnemius muscle (Figure 5C). Ad-irisin ameliorated burn-induced muscle atrophy. Taken together, these results indicate that irisin not only exerted a neuroprotective effect but also rescued motor neuron function and muscle size.

### 2.6. Irisin Attenuated TNFα-Induced Neuronal Damage

**Figure 6:**
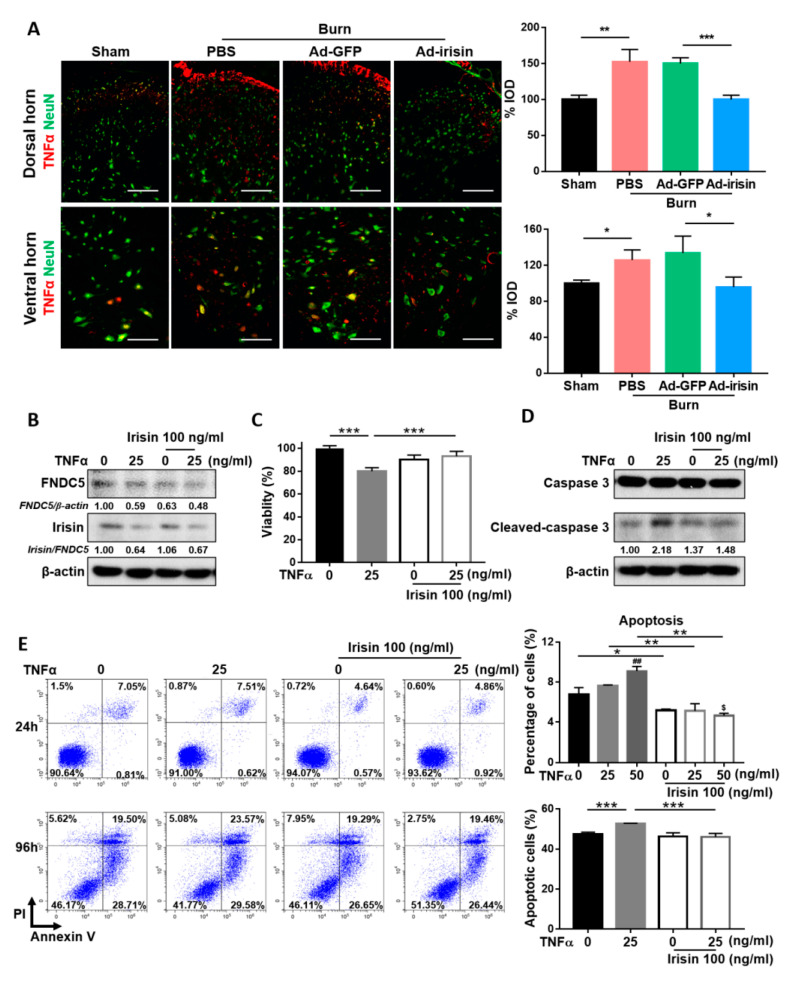
Irisin attenuated TNFα-induced apoptosis in neuronal cells in vivo and in vitro. (**A**) Double immunofluorescence of TNFα and NeuN in the lumbar spinal cord. Representative bar graph illustrating integral optical density of TNFα. Error bars, mean ± SD. * *p* < 0.05, ** *p* < 0.01, *** *p* < 0.001, unpaired *t*-test. Scale bar: 100 μm. SH-SY5Y cells were treated with recombinant irisin and TNFα without serum supplements. Concentrations of irisin and TNFα protein treatments are indicated. (**B**) The cell extract was assayed for FNDC5 and irisin expressions with the 96-h treatment using immunoblotting. The ratios indicate that FNDC5 was normalized to β-actin and that irisin was normalized to FNDC5. (**C**) An MTT assay was conducted for the 96-h treatment (*n* = 14), *** *p* < 0.001. (**D**) Immunoblotting revealed caspase-3 and cleaved caspase-3 for the 96-h treatment. The ratios indicate cleaved-caspase 3/caspase 3 expression. Flow cytometry analysis with double staining of annexin V-FITC and PI was conducted at (**E**) 24 h (top) and 96 h (bottom) of treatment. Bar graphs represent the percentage of the annexin V^+^ population (n = 3). Error bars, mean ± SD. * *p* < 0.05, ** *p* < 0.01, and *** *p* < 0.001; ^##^ *p* < 0.01 compared with the untreated group; ^$^ *p* < 0.05 compared to the irisin 100 ng/mL group, unpaired *t* test.

TNFα is an inflammatory factor that may complicate neuropathy [45]. To evaluate the anti-neuroinflammatory effect of irisin, we examined TNFα expression at 4 weeks postburn. Immunofluorescence staining showed that burn injury resulted in increased TNFα expression, whereas irisin gene delivery attenuated TNFα expression in both the dorsal and ventral horns (Figure 6A). To further investigate the neuroprotective effect of irisin under TNFα stimulus, we evaluated changes in SH-SY5Y cells with irisin and TNFα cotreatment. TNFα treatment impaired the expression of FNDC5 in SH-SY5Y cells (Figure 6B). In addition, TNFα suppressed the cleavage of irisin from FNDC5 and caused a decrease in the irisin/FNDC5 ratio, whereas an attenuated decrease was observed after treatment with irisin (Figure 6B). The MTT assay revealed that irisin rescued SH-SY5Y cells from the TNFα-induced reduction of cell viability (Figure 6C). Further validation analysis indicated that irisin suppressed TNFα-induced expression of cleaved caspase-3 in the 96-h treatment (Figure 6D). Flow cytometry analysis revealed that both the 24- and 96-h irisin treatment alleviated TNFα-induced apoptosis (Figure 6E). An increased overall apoptotic population was observed after the 96-h treatment, which was attributed to serum starvation, a limitation of the in vitro assay (Figure 6E). These results indicate that irisin attenuates TNFα-induced apoptosis in neuronal cells.

## 3. Discussion

By using a third-degree burn rat model [12,17,38], we demonstrated for the first time that burn injury results in FNDC5/irisin downregulation in both the dorsal and ventral horns and decreased irisin levels in the CSF, even after wound healing. Increased neuronal apoptosis was observed in both the dorsal and ventral horns postburn. Intrathecal Ad-irisin restored the levels of spinal FNDC5/irisin and CSF irisin, which ameliorated neuronal damage in the dorsal and ventral horns after burn injury. A mechanical allodynia test confirmed that Ad-irisin attenuated pain sensitization in the ipsilateral hind paw. Examination of the ipsilateral gastrocnemius muscle validated that Ad-irisin restored muscle size. Furthermore, because adenoviral infection with our Ad-irisin system especially infects astrocytes in the nervous system [46], rat C6 cells were able to secret recombinant irisin. The molecular shift in part of the secreted irisin indicated an unknown modification, most likely glycosylation [47]. Double immunofluorescence staining of irisin and GFAP also validated that our Ad-irisin infects astrocytes in vivo. Ad-irisin restored FNDC5/irisin expression in spinal astrocytes postburn. Our in vitro findings demonstrated that irisin exerts neuroprotective effects in both inflammatory signaling and neuronal damage from proinflammatory cytokine stimulation.

Neuropathic pain in postburn scars occurs due to abnormal nerve fiber density, particularly increased dermal innervation with myelinated Aδ fibers or unmyelinated C fibers; however, research has indicated that nerve fiber density is dispensable and that damage or compression of Aδ or C fibers is independent of pain sensitization [6]. Unlike cutaneous innervation, dermal burn injury is associated with neuronal damage and immune response in the spinal cord, which is correlated to neuropathy [12,17,38,48]. Although it is generally accepted that peripheral nerve injury contributes to neuronal apoptosis and is associated with neuropathy, the underlying mechanism remains unknown, and most studies have evaluated peripheral nerve injury using a sciatic nerve injury model [49]. In contrast to sciatic nerve injury, distal damage and burn injury on the skin can result in neuronal damage and subsequent neuropathy. Chang et al. reported that unilateral full-thickness burn injury on the hind paw resulted in prolonged bilateral allodynia, which was associated with increased neuronal excitability and microglial activation in the dorsal horn [48]. Our other studies involving the use of a similar model have demonstrated increased apoptotic neuronal death in both dorsal and ventral horns [12,17,38]. Ma et al. reported that neuronal apoptosis was partially caused by increased expression of inflammatory factors, including IL-1β, IL-10, TNFα, CXCL2, and MCP1, thus inducing microglial activation in the spinal cord [18]. In the present study, we demonstrated that distal damage with third-degree burn injury could also induce neuronal apoptosis in both dorsal and ventral horns. Taken together, these findings suggest that morbidity and severity of neuropathy after burn-induced peripheral nerve injury are strongly associated with spinal neuronal damage.

The dorsal and ventral horns are an essential integrated center for processing and transmitting sensory and motor signals between somata and the brain. Damage to either type of nervous system can cause severe problems, including pain sensitization and physical disability. To date, burn-induced neuropathy has not been fully characterized because of the complex metabolic nature of burn injury, high incidence of sepsis and subsequent use of neurotoxic antibiotics, numerous iatrogenic causes of neuropathy, and interindividual variability in pathophysiology [50]. Furthermore, a complicated interaction network between neurons, microglia, and astrocytes causes a considerable diversity in the causes and severity of burn injuries, making it difficult to determine the relative contribution of different pathways [10], thus precluding definitive diagnosis and therapy. Nevertheless, neuroinflammation is a major pathophysiological cause of neuropathy [28] and can be targeted to alleviate neuropathic pain [51]. According to Ma et al., burn injury results in spinal microglial activation, a marked increase in microglia-induced TNFα expression, and consequently neuronal damage [18]. TNFα is a pain mediator released by M1 microglia/macrophages that promotes neuroinflammation and, subsequently, neuronal damage-related neuropathic pain [45]. Polarization of microglia from the proinflammatory M1 phenotype toward the anti-inflammatory M2 phenotype relieves pain [52]. Irisin promotes the polarization of macrophages toward the M2 phenotype [53]. Similar to macrophages, irisin inhibits microglial activation [25]. In the present study, we demonstrated that recombinant irisin attenuates TNFα-induced apoptosis in SH-Y5Y neuronal cells. TNFα suppresses the cleavage of irisin from its precursor FNDC5 and reduces the irisin/FNDC5 ratio, whereas irisin attenuates this reduction in ratio, suggesting a possible cross-antagonistic effect between TNFα and irisin.

In addition, irisin has regulatory effects on brain-derived neurotropic factor (BDNF) and glial cell-derived neurotropic factor (GDNF) [54,55,56,57]. BDNF is critical in the pathogenesis of chronic pain [58], whereas GDNF attenuates it [59,60]. Some studies have reported that irisin promotes BNDF expression, but this effect was limited to the brain and did not occur in the spinal cord or peripheral nervous system [54,55,56]. BDNF and GDNF are survival-promoting factors in motor neurons [61]. Exercise induces the expression of BNDF in motor neurons, but not in sensory neurons, after peripheral nerve injury [62]. We found that irisin enhanced SMN expression, which is necessary for the survival and neurite outgrowth of motor neurons [40,41]. However, although we demonstrated that irisin attenuated burn-induced damage on motor neurons, further investigating innervation of the neuromuscular junction is warranted to validate burn-related denervation on muscle. In addition, aerobic exercise training relieves neuropathy by reducing BDNF levels in dorsal root ganglion following peripheral nerve injury [63] and normalizes spinal GDNF levels following spinal cord injury [64]. Taken together, these findings indicate that irisin has distinct effects on BDNF/GDNF levels, depending on central/peripheral sensitization and the neuronal type. Further investigation of the role of irisin in BDNF/GDNF regulation on sensory or motor neurons is required. In addition, irisin expression is enriched in GABAergic cells [65], which release gamma-aminobutyric acid (GABA), a major inhibitory neurotransmitter, which inhibits neuropathic pain [66,67,68]. Exercise increases circulating GABA [69] and prevents a reduction in the level of glutamate decarboxylase 65, which catalyzes the decarboxylation of glutamate to GABA in the nervous system [35]. Although several studies have revealed that exercise promotes GABA signaling [70,71,72], how irisin regulates the GABA pathway remains unclear. In addition, most studies investigating the neurofunctions of irisin have been conducted on the brain system and not on the spinal cord or peripheral nervous system. Further studies should investigate the roles of irisin and neurotrophic factors in the spinal cord or peripheral nervous system.

## 4. Materials and Methods

### 4.1. Animals

Adult male Sprague Dawley rats were obtained from BioLASCO Taiwan (Taipei, Taiwan). All rats were maintained in specific pathogen-free animal facilities with water and commercial rat food provided ad libitum under a 12/12-h light/dark cycle. Our experimental design was approved by the Institutional Animal Care and Use Committee of Kaohsiung Medical (approval numbers: 106192 and 107200).

### 4.2. Cell Culture

Rat C6 and human SH-SY5Y cells were obtained from American Type Culture Collection and grown under humidified conditions in 5% CO_2_ at 37 °C with a supplement of 10% heat-inactivated serum. Treatment of recombinant proteins was applied following serum starvation overnight in SH-SY5Y cells. Adenoviral infection was manipulated for 48-h infection in C6 cells. The collected culture medium was centrifuged at 20,000× *g* to exclude debris. Both cell extract and culture medium were stored at −20 °C until analysis.

### 4.3. Adenovirus

The vector used for the generation of Ad-irisin was designed using an AdEasy adenoviral vector system (Agilent Technologies) as previously described [73]. Complete irisin cDNA with an N-terminal signal sequence was obtained from the complete FNDC5 cDNA of *Mus musculus*, followed by amplification through polymerase chain reaction (PCR) with two primers: 5′ CGCCGGCGATGCCCCCAGGGCCGTGC 3′ and 5′ CTCGAGCTCCTTCATGGTCACCTC 3′. For Ad-irisin production, the recombinant irisin cDNA was subcloned into multiple cloning sites on a pShuttle-IRES-hrGFP-2 vector. The pShuttle vector was then recombined with the pAdEasy-1 vector in BJ5183-competent cells to derive complete adenoviral cDNA. After the transfection of recombinant adenoviral cDNA into HEK293 cells, the adenovirus was selected with an adenovirus plaque assay and amplified in HEK293 cells.

### 4.4. Recombinant Proteins

Recombinant irisin was produced using our developed system [74]. Irisin cDNA was amplified using PCR and subcloned into the pET28a vector (Novagen, Madison, WI, USA). The pET28a–irisin plasmid was then transformed into BL21-competent cells. The recombinant protein was collected following stimulation with isopropyl β- d-1-thiogalactopyranoside After endotoxin elimination with a DetoxiGel column (Pierce Biotechnology, Waltham, MA, USA), the purity and molecular weight of the recombinant protein were examined through Coomassie blue staining and immunoblotting analysis following SDS-PAGE electrophoresis. Recombinant TNFα was obtained from PEPROTECH^®^ (Rehovot, Israel).

### 4.5. Burn Injury Model and Mechanical Allodynia Test

The burn injury model and mechanical allodynia test were employed as previously described [17]. In brief, third-degree burn injury was induced in adult male Sprague Dawley rats, weighing 160–180 g. After the induction of anesthesia with Zoletil 50 (50 μg/g; Virbac Laboratories, Carros, France), the right hind paw was placed on a heated metal block (75 °C ± 0.5 °C) with a 100-g object on the top of the paw to maintain a constant contact between the plantar surface of the paw and the metal block for 10 s. Silver sulfadiazine cream was applied to the burn paw for approximately 21 days until their wounds healed. The mean mechanical allodynia threshold was derived from triplicate measurements once daily using Dynamic Plantar Aesthesiometer (Ugo Basile, Gemonio, Italy) before the rats were sacrificed. The L4–L6 spinal cord and CSF were harvested at 4 weeks postburn and stored at −80 °C until analysis.

### 4.6. Intrathecal Injections

Intrathecal injections were administered at 3 weeks postburn. A polyurethane catheter was placed in the intrathecal space, reaching the L4–L6 segments. Following examination of leakage and permeability, the free end of the catheter was sealed and placed subcutaneously 1 day before injection. After overnight recovery, the rats without significant surgical influence were randomly assigned to groups, and adenovirus was injected into the implanted catheter. PBS and Ad-GFP were used as the injection control and negative control, respectively.

### 4.7. Immunofluorescence and H&E Staining

For immunofluorescence staining, frozen tissues were sliced to 5 μm and permeabilized using a buffer containing 1.5% normal serum with 0.2% Triton X-100 in TBST. Following incubation with specific antibodies, the sample was mounted with antifade media and visualized under a fluorescence microscope. TUNEL staining was performed in accordance with the manufacturer’s instructions (S7110; Merck Millipore, Billerica, MA, USA). TUNEL^+^ cells were counted in 3 fields for each tissue section of each rat in each group. For hematoxylin and eosin (H&E) staining, 5-μm-thick paraffin-embedded tissue sections were first cut, dewaxed, and rehydrated. After being completely washed, the tissue was dehydrated, sealed with mounting media, and assessed under a microscope. The area of myofiber was obtained from an average area in 3 fields for each muscle section of each rat.

### 4.8. Immunoblotting Analysis and Irisin ELISA

Tissues and cells were homogenized in radioimmunoprecipitation assay buffer containing protease and phosphatase inhibitors (Roche, Basel, Switzerland). Protein extracts were heat-denatured and separated with electrophoresis using SDS-PAGE (Bio-Rad, Burlington, MA, USA) and transferred onto a polyvinylidene fluoride transfer membrane. Following blocking with 5% skim milk, the membrane was incubated with a specific primary antibody and HRP-conjugated secondary antibody. Signals were visualized and exposed on an X-ray film (Fujifilm Corporation, Kyoto, Japan). The signal density and intensity were determined using ImageJ software (NIH, Bethesda, MD, USA). The CSF irisin level was detected using an irisin detection kit according to the manufacturer’s instructions (Phoenix Pharmaceuticals, Burlingame, CA, USA).

### 4.9. Flow Cytometry Analysis

Cells were isolated, washed twice, and subsequently stained with annexin V-FITC and PI according to the manufacturer’s instructions (BioLegend, San Diego, CA, USA). All cells were analyzed with a flow cytometry analyzer (Beckman, Brea, CA, USA) immediately without fixation.

### 4.10. Statistics

Student’s *t*-test was used to identify differences. All values are expressed as the mean ± standard deviation, and *p* < 0.05 was considered statistically significant.

## 5. Conclusions

**Figure 7:**
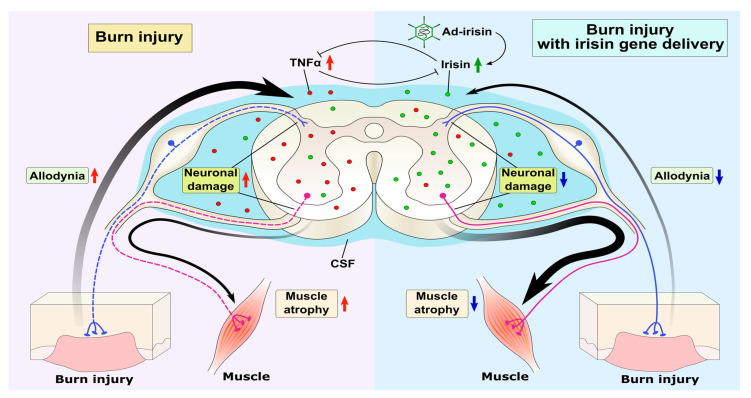
Graphic summary of irisin-attenuated neuropathies. Peripheral third-degree burn injury increased the expression of TNFα in the spinal cord, thus reducing the irisin level and neuronal damage in both the dorsal and ventral horns. Adenovirus containing the irisin sequence restored spinal FNDC5/irisin and CSF irisin levels, thus antagonizing the TNFα-induced inflammatory response and subsequent neuronal damage in both the dorsal and ventral horns and ameliorating allodynia and muscle atrophy.

We demonstrated that third-degree burn injury can result in neuronal damage in both dorsal and ventral horns, leading to allodynia and denervation muscle atrophy. Intrathecal Ad-irisin injection restored the expression of FNDC5/irisin and TNFα in the lumbar spinal cord, as well as irisin level in CSF, thereby alleviating neuroinflammation and preventing neuronal apoptosis. These results suggest that spinal FNDC5/irisin and CSF irisin are crucial for the survival of spinal neurons and consequent pain sensitization and muscle atrophy after burn injury. Further investigation is warranted to elucidate the mechanism of irisin in neural immunity and neurotrophic regulation (Figure 7).

## Supplementary Materials

Supplementary materials can be found at https://www.mdpi.com/1422-0067/21/20/7798/s1, Figure S1: Irisin gene delivery restored decreased FNDC5/irisin expression in astrocytes postburn. Double immunofluorescence staining of irisin and GFAP in the dorsal and ventral horns of L4–L6 segments at 4 weeks postburn. Arrowheads indicate double-positive cells. Representative bar graph illustrating the ratio of irisin^+^GFAP^+^ to GFAP^+^ cells. Error bars, mean ± SD. * *p* < 0.05, ** *p* < 0.01, *** *p* < 0.001, unpaired *t* test. Scale bar: 100 μm.

## Abbreviations

FDNC5	Fibronectin Type III Domain Containing 5
TNFα	Tumor necrosis factor alpha
CSF	Cerebrospinal fluid
NeuN	Neuronal nuclei
SMN	Survival motor neuron
ChAT	Choline acetyltransferase
IL-6	Interleukin 6
IL-10	Interleukin 10
IL-1β	Interleukin 1 beta
NF-κB	Nuclear Factor Kappa B
Iba1	Ionized calcium-binding adaptor molecule 1
MPO-1	Myeloperoxidase-1
MCP1	Monocyte chemoattractant protein-1
CXCL2	C-X-C Motif Chemokine Ligand 2
GFAP	Glial fibrillary acidic protein
BDNF	Brain-derived neurotrophic factor
GDNF	Glial cell-derived neurotrophic factor
COX-2	Cyclooxygenase-2
PGE_2_	Prostaglandin E2
PGI_2_	Prostaglandin I2
GABA	γ-Aminobutyric acid
MOI	Multiplicity of infection
HA	Hemagglutinin
GFP	Green fluorescent protein

## References

[1] Marquez S., Turley J.J.E., Peters W.J. (1993). Neuropathy in burn patients. Brain.

[2] Vetrichevvel T.P., Randall S.M., Fear M.W., Wood F.M., Boyd J.H., Duke J.M. (2016). Burn injury and long-term nervous system morbidity: A population-based cohort study. BMJ Open.

[3] Tamam Y., Tamam C., Tamam B., Ustundag M., Orak M., Tasdemir N. (2013). Peripheral neuropathy after burn injury. Eur. Rev. Med. Pharmacol. Sci..

[4] Klein M.B., Hollingworth W., Rivara F.P., Kramer C.B., Askay S.W., Heimbach D.M., Gibran N.S. (2008). Hospital Costs Associated With Pediatric Burn Injury. J. Burn. Care Res..

[5] Malenfant A., Forget R., Amsel R., Papillon J., Frigon J.-Y., Choinière M. (1998). Tactile, thermal and pain sensibility in burned patients with and without chronic pain and paresthesia problems. Pain.

[6] Bijlard E. (2017). A Systematic Review on the Prevalence, Etiology, and Pathophysiology of Intrinsic Pain in Dermal Scar Tissue. Pain Physician.

[7] Strong A.L., Agarwal S., Cederna P.S., Levi B. (2017). Peripheral Neuropathy and Nerve Compression Syndromes in Burns. Clin. Plast. Surg..

[8] Alfonso-Sanchez J.L., Pereperez S.B., Bastida J., Martínez M. (2007). Cost-Utility Analysis Applied to the Treatment of Burn Patients in a Specialized Center. Arch. Surg..

[9] Ji Q., Jia H., Dai H., Li W., Zhang L. (2010). Protective effects of pentoxifylline on the brain following remote burn injury. Burns.

[10] Morgan M., Deuis J.R., Froesig-Joergensen M., Lewis R.J., Cabot P.J., Gray P., Vetter I. (2017). Burn Pain: A Systematic and Critical Review of Epidemiology, Pathophysiology, and Treatment. Pain Med..

[11] Evers L.H., Bhavsar D., Mailänder P. (2010). The biology of burn injury. Exp. Dermatol..

[12] Lin C.-H., Wu S.-H., Lee S.-S., Lin Y.-N., Kuo Y.-R., Chai C.-Y., Huang S.-H. (2017). Autologous Adipose-Derived Stem Cells Reduce Burn-Induced Neuropathic Pain in a Rat Model. Int. J. Mol. Sci..

[13] Schneider J.C., Harris N.L., El Shami A., Sheridan R.L., Schulz J.T., Bilodeau M.-L., Ryan C.M. (2006). A Descriptive Review of Neuropathic-Like Pain After Burn Injury. J. Burn. Care Res..

[14] Cavalli E., Mammana S., Nicoletti F., Bramanti P., Mazzon E. (2019). The neuropathic pain: An overview of the current treatment and future therapeutic approaches. Int. J. Immunopathol. Pharmacol..

[15] Wu C., Calvert C.T., Cairns B.A., Hultman C.S. (2013). Lower Extremity Nerve Decompression in Burn Patients. Ann. Plast. Surg..

[16] Higashimori H., Whetzel T.P., Mahmood T., Carlsen R.C. (2005). Peripheral axon caliber and conduction velocity are decreased after burn injury in mice. Muscle Nerve.

[17] Wu S.-H., Huang S.-H., Cheng K.-I., Chai C.-Y., Yeh J.-L., Wu T.-C., Hsu Y.-C., Kwan A.-L. (2015). Third-Degree Hindpaw Burn Injury Induced Apoptosis of Lumbar Spinal Cord Ventral Horn Motor Neurons and Sciatic Nerve and Muscle Atrophy in Rats. BioMed Res. Int..

[18] Ma L., Zhou Y., Khan M.A.S., Yasuhara S., Martyn J.A.J. (2019). Burn-Induced Microglia Activation is Associated With Motor Neuron Degeneration and Muscle Wasting in Mice. Shock.

[19] Bostroem P., Wu J., Jedrychowski M.P., Korde A., Ye L., Lo J.C., Rasbach K.A., Bostroem E.A., Choi J.H., Long J.Z. (2012). A PGC1-α-dependent myokine that drives brown-fat-like development of white fat and thermogenesis. Nat. Cell Biol..

[20] Arhire L.I., Mihalache L., Covasa M. (2019). Irisin: A Hope in Understanding and Managing Obesity and Metabolic Syndrome. Front. Endocrinol..

[21] Perakakis N., Triantafyllou G.A., Fernández-Real J.M., Huh J.Y., Park K.H., Seufert J., Mantzoros C.S. (2017). Physiology and role of irisin in glucose homeostasis. Nat. Rev. Endocrinol..

[22] Gizaw M., Anandakumar P., Debela T. (2017). A Review on the Role of Irisin in Insulin Resistance and Type 2 Diabetes Mellitus. J. Pharmacopunct..

[23] Islam M.R., Young M.F., Wrann C.D., Spiegelman B. (2017). The Role of FNDC5/Irisin in the Nervous System and as a Mediator for Beneficial Effects of Exercise on the Brain. Hormones, Metabolism and the Benefits of Exercise.

[24] Mazur-Bialy A.I., Pochec E., Zarawski M. (2017). Anti-Inflammatory Properties of Irisin, Mediator of Physical Activity, Are Connected with TLR4/MyD88 Signaling Pathway Activation. Int. J. Mol. Sci..

[25] Li D.-J., Li Y.-H., Yuan H.-B., Qu L.-F., Wang P. (2017). The novel exercise-induced hormone irisin protects against neuronal injury via activation of the Akt and ERK1/2 signaling pathways and contributes to the neuroprotection of physical exercise in cerebral ischemia. In Metabolism: Clinical and experimental. Metabolism.

[26] Wang K., Li H., Wang H., Wang J.-H., Song F., Sun Y. (2018). Irisin Exerts Neuroprotective Effects on Cultured Neurons by Regulating Astrocytes. Mediat. Inflamm..

[27] Jin Z., Guo P., Li X., Ke J., Wang Y., Wu H. (2019). Neuroprotective effects of irisin against cerebral ischemia/reperfusion injury via Notch signaling pathway. Biomed. Pharmacother..

[28] Ellis A., Bennett D.L.H. (2013). Neuroinflammation and the generation of neuropathic pain. Br. J. Anaesth..

[29] Skaper S.D., Facci L., Zusso M., Giusti P. (2017). Neuroinflammation, Mast Cells, and Glia: Dangerous Liaisons. Neuroscientist.

[30] Chen Y.-W., Li Y.-T., Li Z.-Y., Hung C.-H. (2012). Exercise Training Attenuates Neuropathic Pain and Cytokine Expression After Chronic Constriction Injury of Rat Sciatic Nerve. Anesth. Analg..

[31] Dobson J.L., McMillan J., Li L. (2014). Benefits of exercise intervention in reducing neuropathic pain. Front. Cell. Neurosci..

[32] Kelly A.M. (2018). Exercise-Induced Modulation of Neuroinflammation in Models of Alzheimer’s Disease. Brain Plast..

[33] Ignácio Z.M., Da Silva R.S., Plissari M.E., Quevedo J., Réus G.Z. (2019). Physical Exercise and Neuroinflammation in Major Depressive Disorder. Mol. Neurobiol..

[34] Dameni S., Janzadeh A., Yousefifard M., Nasirinezhad F. (2018). The effect of intrathecal injection of irisin on pain threshold and expression rate of GABAB receptors in peripheral neuropathic pain model. J. Chem. Neuroanat..

[35] Farzad B., Rajabi H., Gharakhanlou R., Allison D.J., Hayat P., Jameie S.B. (2018). Swimming Training Attenuates Allodynia and Hyperalgesia Induced by Peripheral Nerve Injury in an Adult Male Rat Neuropathic Model: Effects on Irisin and GAD65. Pain Med..

[36] Saraç F., Sarsu S.B., Yeniocak S., Şahin K., Yucetas E., Yildirim D., Koldas M., Uzun O. (2018). The Diagnostic Value of Irisin in Pediatric Patients with Acute Abdominal Pain. Emerg. Med. Int..

[37] Orellana C., Calvet J., Navarro N., Galisteo C., Gratacós J., Larrosa M. (2018). AB0962 Irisin levels are associated with exercise, pain and function in patients with knee osteoarthritis. Osteoarthritis.

[38] Huang S.-H., Wu S.-H., Lee S.-S., Chang K.-P., Chai C.-Y., Yeh J.-L., Lin S.-D., Kwan A.-L., Wang H.-M.D., Lai C.-S. (2015). Fat Grafting in Burn Scar Alleviates Neuropathic Pain via Anti-Inflammation Effect in Scar and Spinal Cord. PLoS ONE.

[39] Decosterd I., Woolf C.J. (2000). Spared nerve injury: An animal model of persistent peripheral neuropathic pain. Pain.

[40] Simon C.M., Janas A.M., Lotti F., Tapia J.C., Pellizzoni L., Mentis G.Z. (2016). A Stem Cell Model of the Motor Circuit Uncouples Motor Neuron Death from Hyperexcitability Induced by SMN Deficiency. Cell Rep..

[41] Fan L., Simard L.R. (2002). Survival motor neuron (SMN) protein: Role in neurite outgrowth and neuromuscular maturation during neuronal differentiation and development. Hum. Mol. Genet..

[42] Chaytow H., Huang Y.-T., Gillingwater T.H., Faller K.M.E. (2018). The role of survival motor neuron protein (SMN) in protein homeostasis. Cell. Mol. Life Sci..

[43] Laird A.S., Mackovski N., Rinkwitz S., Becker T.S., Giacomotto J. (2016). Tissue-specific models of spinal muscular atrophy confirm a critical role of SMN in motor neurons from embryonic to adult stages. Hum. Mol. Genet..

[44] Soler-Botija C., Cuscó I., Lopez E., Clua A., Saladich I.G., Baiget M., Ferrer I., Tizzano E.F. (2005). Choline acetyltransferase expression does not identify early pathogenic events in fetal SMA spinal cord. Neuromuscul. Disord..

[45] Leung L., Cahill C.M. (2010). TNF-alpha and neuropathic pain—A review. J. Neuroinflamm..

[46] Merienne N., Le Douce J., Faivre E., Déglon N., Bonvento G. (2013). Efficient gene delivery and selective transduction of astrocytes in the mammalian brain using viral vectors. Front. Cell. Neurosci..

[47] Nie Y., Liu D. (2017). N-Glycosylation is required for FDNC5 stabilization and irisin secretion. Biochem. J..

[48] Chang Y.-W., Tan A., Saab C., Waxman S.G. (2010). Unilateral Focal Burn Injury Is Followed by Long-Lasting Bilateral Allodynia and Neuronal Hyperexcitability in Spinal Cord Dorsal Horn. J. Pain.

[49] Liu Y., Wang H. (2019). Peripheral nerve injury induced changes in the spinal cord and strategies to counteract/enhance the changes to promote nerve regeneration. Neural Regen. Res..

[50] Kowalske K., Holavanahalli R., Helm P. (2001). Neuropathy After Burn Injury. J. Burn Care Rehabil..

[51] Ji R.-R., Xu Z.-Z., Gao Y.-J. (2014). Emerging targets in neuroinflammation-driven chronic pain. Nat. Rev. Drug Discov..

[52] Huo W., Zhang Y., Liu Y., Lei Y., Sun R., Zhang W., Huang Y., Mao Y., Wang C., Ma Z. (2018). Dehydrocorydaline attenuates bone cancer pain by shifting microglial M1/M2 polarization toward the M2 phenotype. Mol. Pain.

[53] Ye W., Wang J., Lin D., Ding Z. (2020). The immunomodulatory role of irisin on osteogenesis via AMPK-mediated macrophage polarization. Int. J. Biol. Macromol..

[54] Wrann C.D., White J.P., Salogiannnis J., Laznik-Bogoslavski D., Wu J., Ma D., Lin J.D., Greenberg M.E., Spiegelman B.M. (2013). Exercise induces hippocampal BDNF through a PGC-1alpha/FNDC5 pathway. Cell Metab..

[55] Chen J.T.-C., Guo D., Campanelli D., Frattini F., Mayer F., Zhou L., Kuner R., Heppenstall P.A., Knipper M., Hu J. (2014). Presynaptic GABAergic inhibition regulated by BDNF contributes to neuropathic pain induction. Nat. Commun..

[56] Kim O.Y., Song J. (2018). The Role of Irisin in Alzheimer’s Disease. J. Clin. Med..

[57] Wahab F., Drummer C., Mätz-Rensing K., Fuchs E., Behr R. (2020). Irisin is expressed by undifferentiated spermatogonia and modulates gene expression in organotypic primate testis cultures. Mol. Cell. Endocrinol..

[58] Sikandar S., Minett M.S., Millet Q., Santana-Varela S., Lau J., Wood J.N., Zhao J. (2018). Brain-derived neurotrophic factor derived from sensory neurons plays a critical role in chronic pain. Brain.

[59] Boucher T.J., Okuse K., Bennett D.L.H., Munson J.B., Wood J.N., McMahon S.B. (2000). Potent Analgesic Effects of GDNF in Neuropathic Pain States. Science.

[60] Takasu K., Sakai A., Hanawa H., Shimada T., Suzuki H. (2011). Overexpression of GDNF in the Uninjured DRG Exerts Analgesic Effects on Neuropathic Pain Following Segmental Spinal Nerve Ligation in Mice. J. Pain.

[61] Yan Q., Elliott J., Snider W.D. (1992). Brain-derived neurotrophic factor rescues spinal motor neurons from axotomy-induced cell death. Nat. Cell Biol..

[62] Keeler B.E., Liu G., Siegfried R.N., Zhukareva V., Murray M., Houlé J.D. (2012). Acute and prolonged hindlimb exercise elicits different gene expression in motoneurons than sensory neurons after spinal cord injury. Brain Res..

[63] Cobianchi S., Casals-Diaz L., Jaramillo J., Navarro X. (2013). Differential effects of activity dependent treatments on axonal regeneration and neuropathic pain after peripheral nerve injury. Exp. Neurol..

[64] Detloff M.R., Smith E.J., Molina D.Q., Ganzer P.D., Houlé J.D. (2014). Acute exercise prevents the development of neuropathic pain and the sprouting of non-peptidergic (GDNF- and artemin-responsive) c-fibers after spinal cord injury. Exp. Neurol..

[65] Dun S.L., Lyu R.-M., Chen Y.-H., Chang J.-K., Luo J.J., Dun N.J. (2013). Irisin-immunoreactivity in neural and non-neural cells of the rodent. Neuroscience.

[66] Hwang J.H., Yaksh T.L. (1997). The effect of spinal GABA receptor agonists on tactile allodynia in a surgically-induced neuropathic pain model in the rat. Pain.

[67] Braz J.M., Sharif-Naeini R., Vogt D., Kriegstein A., Alvarez-Buylla A., Rubenstein J.L., Basbaum A.I. (2012). Forebrain GABAergic Neuron Precursors Integrate into Adult Spinal Cord and Reduce Injury-Induced Neuropathic Pain. Neuron.

[68] Li C., Lei Y., Tian Y., Xu S., Shen X., Wu H., Bao S., Wang F. (2019). The etiological contribution of GABAergic plasticity to the pathogenesis of neuropathic pain. Mol. Pain.

[69] Roberts L.D., Ashmore T., McNally B.D., Murfitt S.A., Fernandez B.O., Feelisch M., Lindsay R., Siervo M., Williams E.A., Murray A.J. (2017). Inorganic nitrate mimics exercise-stimulated muscular fiber-Type switching and myokine and g-Aminobutyric acid release. Diabetes.

[70] Pour M.B., Bayat M., Golab F., Eftekharzadeh M., Katebi M., Soleimani M., Karimzadeh F. (2019). The effect of exercise on GABA signaling pathway in the model of chemically induced seizures. Life Sci..

[71] Coxon J.P., Cash R.F.H., Hendrikse J.J., Rogasch N.C., Stavrinos E., Suo C., Yücel M. (2017). GABA concentration in sensorimotor cortex following high-intensity exercise and relationship to lactate levels. J. Physiol..

[72] Maddock R.J., Casazza G.A., Fernandez D.H., Maddock M.I. (2016). Acute Modulation of Cortical Glutamate and GABA Content by Physical Activity. J. Neurosci..

[73] Tai M. (2003). Gene transfer of glial cell line-derived neurotrophic factor promotes functional recovery following spinal cord contusion. Exp. Neurol..

[74] Hu T.-H., Huang C.-C., Liu L.-F., Lin P.-R., Liu S.-Y., Chang H.-W., Changchien C.-S., Lee C.-M., Chuang J.-H., Tai M.H. (2003). Expression of hepatoma-derived growth factor in hepatocellular carcinoma. Cancer.

