# Supplementary material for: Irisin Gene Delivery Ameliorates Burn-Induced Sensory and Motor Neuropathy
Source: Int J Mol Sci. 2020 Oct 21;21(20):7798. doi: 10.3390/ijms21207798 (PMC7589574; doi:10.3390/ijms21207798)
Supplement: Supplementary file 1 [file ijms-21-07798-s001.pdf]

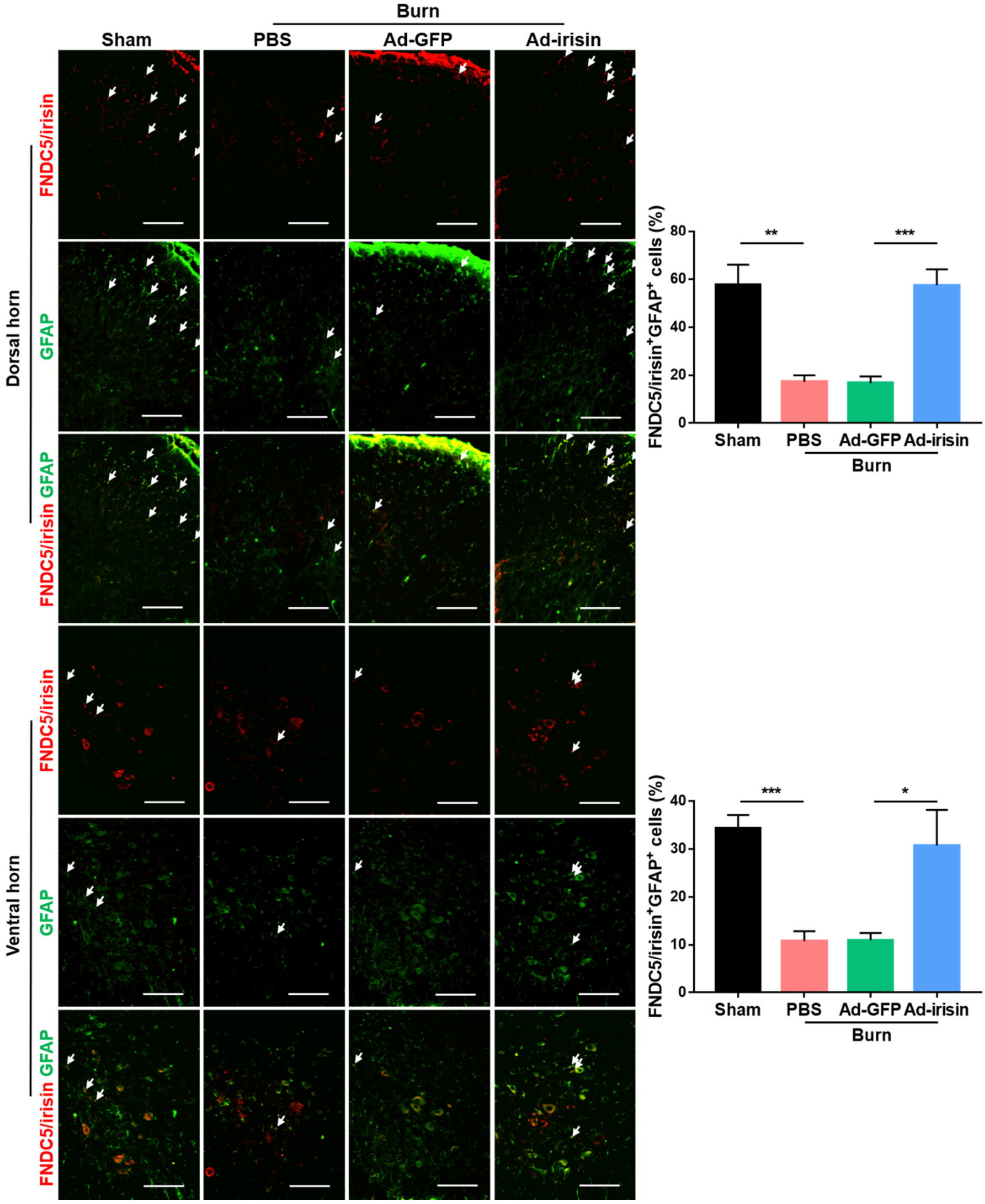


**Figure S1.** Irisin gene delivery restored decreased FNDC5/irisin expression in astrocytes postburn. Double immunofluorescence staining of irisin and GFAP in the dorsal and ventral horns of L4–L6 segments at 4 weeks postburn. Arrowheads indicate double-positive cells. Representative bar graph illustrating the ratio of irisin$^+$GFAP$^+$ to GFAP$^+$ cells. Error bars, mean ± SD. $*p < 0.05$, $**p < 0.01$, $***p < 0.001$, unpaired *t* test. Scale bar: 100 μm.
